# Polymorphisms of Antioxidant Genes as a Target for Diabetes Management

**DOI:** 10.22088/acadpub.BUMS.6.3.135

**Published:** 2017-08-14

**Authors:** Ozra Tabatabaei-Malazy, Mehrnoosh Khodaeian, Fatemeh Bitarafan, Bagher Larijani, Mahsa M.Amoli

**Affiliations:** 1 *Diabetes Research Center, Endocrinology and Metabolism Clinical Sciences Institute, Tehran University of Medical Sciences, Tehran, Iran.*; 2 *Endocrinology and Metabolism Research Center, Endocrinology and Metabolism Clinical Sciences Institute, Tehran University of Medical Sciences, Tehran, Iran.*; 3 *Metabolic Disorders Research Center, Endocrinology and Metabolism Molecular -Cellular Sciences Institute, Tehran University of Medical Sciences, Tehran, Iran.*

**Keywords:** Diabetes mellitus, oxidative stress, antioxidants, polymorphism

## Abstract

Diabetes mellitus (DM) is one of the most important health problems with increasing prevalence worldwide. Oxidative stress, a result of imbalance between reactive oxygen species (ROS) generation and antioxidant defense mechanisms has been demonstrated as the main pathology in DM. Hyperglycemia-induced ROS productions can induce oxidative stress through four major molecular mechanisms including the polyol pathway, advanced glycation end- products formation, activation of protein kinase C isoforms, and the hexosamine pathways. In the development of type 2 DM (T2DM) and its complications, genetic and environmental factors play important roles. Therefore, the aim of this review was to focus on the assessment of single-nucleotide polymorphisms within antioxidant enzymes including superoxide dismutase, catalase, glutathione peroxidase, glutathione-S-transferase, nitric oxide synthase, and NAD(P)H oxidase and their association with T2DM. The results would be helpful in understanding the mechanisms involved in pathogenesis of disease besides discovering new treatment approaches in management of DM.

Diabetes mellitus (DM) as the major epidemic disorder of the current century is a multifactorial condition and is influenced by both genetic background and environmental factors (1-3). According to International Diabetes Federation (IDF) report in 2015, there are 415 million diabetic patients worldwide. This number is expected to reach 642 million by 2040 (4).

Several lines of evidence suggest that oxidative stress plays a pivotal role in the pathogenesis of a wide range of human disorders including cancers, diabetes, cardiovascular disorders, kidney diseases, and neurodegenerative diseases (5-11). In addition, the critical pathogenic role of oxidative stress in the initiation and development of diabetes complications has been determined (12). Generation of reactive oxygen species (ROS) secondary to hyperglycemia may lead to increased oxidative stress in β-cells which cause β-cell dysfunction and other long-term complications of diabetes because of insulin secretion and/or its function impairment (5,13).

Oxidative stress is defined as a disruption in balance between ROS and antioxidants produced upon oxidative damage (8,11-14). ROS includes a series of oxygen intermediates such as superoxide anion, hydrogen peroxide, hydroxyl radical, and hypochlorous acid (15). Although under normal physiological conditions, ROS could help in cell defense, hormone synthesis, signal transduction, transcription factor regulation, and gene expression, but under pathological conditions aberrant tissue damage, inflammation, fibrosis, and β-cell death can occur (13). The four major mechanisms involved in increased intracellular oxidative stress as a result of hyperglycemia, are polyol pathway, advanced glycation end-products (AGEs), protein kinase C (PKC)-diacylglycerol (DAG) and the hexosamine pathways (9,16). It has been shown that all of these pathways are activated by mitochondrial ROS overproduction (9) (Figure 1). The effects of ROS can be modified by enzymatic or non-enzymatic antioxidants. Enzymatic antioxidants include superoxide dismutase (SOD), catalase (CAT), glutathione peroxidase (GPx), glutathione-S-transferase (GST), nitric oxide synthase (NOS), and nicotinamide adenine dinucleotide phosphate (NADPH) oxidase (NOX) (2,3,10-13), and non-enzymatic antioxidants consist of vitamins, minerals, carotenoids, polyphenols, and some other molecules (17-19) (Figure 1). 

**Fig 1:**
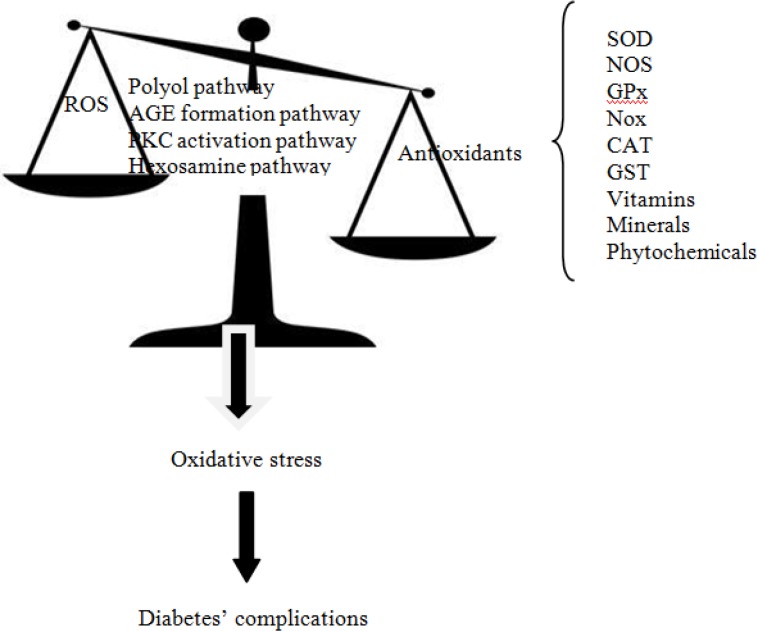
Pathways influencing diabetes in association between reactive oxygen species and antioxidants. ROS: reactive oxygen species; SOD: superoxide dismutase; NOS: nitric oxide synthase; GPx: glutathione peroxidase; Nox: NAD(P)H oxidase; CAT: catalase; GST: glutathione-S-transferase; AGE: advanced glycation end-products, PKC: protein kinase C.

Moreover, other protectors against oxidative stress have been demonstrated including uncoupling proteins (UCP), cyclooxygenase (COX), and paraoxonase (PON) gene families that have anti-oxidative properties, and therefore seem to play central role in diabetes. It has been shown that the single-nucleotide polymorphisms (SNPs) of these antioxidant enzymes are involved in the pathogenesis of diabetes and its complications (2,10). 

However, in this paper we aimed to review the evidence on main pathways involved in oxidative stress and the polymorphisms of antioxidant enzymes including SOD, CAT, GPx, GST, NOS, and NOX in association with type 2 DM (T2DM).

## Major mechanisms of hyperglycemia-induced damage

***Polyol pathway***

**Fig 2:**
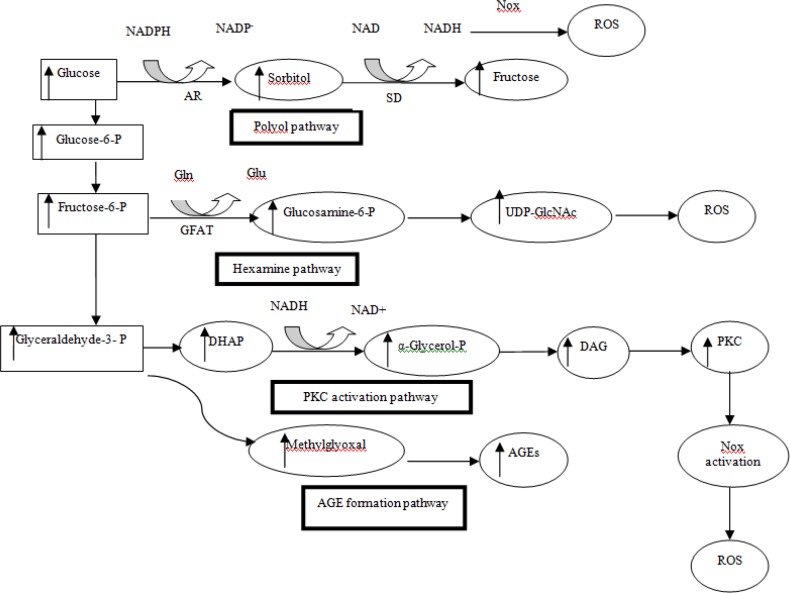
Hyperglycemic-induced oxidative stress pathways. NADPH, nicotinamide adenine dinucleotide phosphate; AR, Aldose reductase; SD, Sorbitol dehydrogenase; Nox, NAD(P)H oxidase; Gln, glutamine; Glu, glutamic acid; GFAT, Glutamine fructose-6-phosphate amidotransferase; UDP-GlcNAc, Uridine diphosphate N-acetylglucosamine; DHAP, Dihydroxyacetone phosphate; DAG, diacylglycerol; PKC, protein kinase C; AGE, advanced glycation end-products, ROS, reactive oxygen species.

The polyol pathway uses NADPH and converts excessive intracellular glucose into forms of sugar alcohols through aldose reductase enzyme. In non-diabetic subjects, this pathway utilizes very small fraction of total glucose. But in diabetics, aldose reductase is activated and induces increasing conversion of glucose to sorbitol. Then, sorbitol is oxidized to fructose by enzyme sorbitol dehydrogenase (SDH) with NAD^+^ as a cofactor. Consumption of NADPH reduces glutathione (GSH) reductase activity that its regeneration is dependent on NADPH. On the other hand, GSH is known as an important scavenger of ROS. Finally, this process induces ROS and exacerbates intracellular oxidative stress (9,12,14,16)(Figure 2).

In the literature, it was shown that over-expression of aldose reductase in diabetic mice resulted in increased atherosclerosis and reduction of glutathione [9]. Also, in an experiment performed in diabetic rats’ eyes, reduction of GSH was observed in their lens due to over-expression of aldose reductase (9). It was reported that reduction in nitric oxide (NO) availability might result in reduction of cellular glutathionylation and therefore inducing ROS production in diabetic rats (9).

***Intracellular AGEs formation pathway***

Hyperglycemia induces overproduction of both extracellular and intracellular AGEs. AGEs are the result of glyoxal oxidation, 3-deoxyglucoson formation, and fragmentation of glyceraldehyde-3-phosphate into methylglyoxal (15). Cell damage occurs due to intracellular production of AGE precursors through 3 general mechanisms. At first, the function of intracellular proteins modifications by AGEs is altered. Then, abnormal interaction of extracellular matrix components modified by AGE precursors with other matrix components and with matrix receptors such as integrins is observed. Finally, plasma proteins are modified by AGEs precursors binding to cell surface receptors such as receptor for AGEs (RAGE), or macrophage scavenger receptors. Generation of ROS and activation of nuclear factor-κB (NF-κB) may be initiated by AGE-RAGE interaction via cytosolic-NADPH oxidase-dependent mechanisms. AGE-RAGE interaction may also contribute to oxidative stress via the induction of mitochondrial superoxide (1, 9, 16, 20) (Figure 2).

These data suggest that in diabetes, the increased production of AGEs might alter glucose metabolism through direct attack on pancreatic insulin-producing cells (20).

Increased concentrations of RAGE in diabetic individuals has been demonstrated in different studies. Transgenic mice with over-expression of RAGE showed accelerated kidney disease, and conversely, administration of RAGE neutralizing antibodies in rodent models of diabetes has revealed protection against renal complications (1). Increased AGEs level contributes to increased rate of lower limb amputation, heart failure, and mortality after ischemic events which seems to be mediated by reduction in the number of collateral vessels in angiogram of diabetic patients (9).

In a cohort of diabetes and atherosclerosis in Maastricht (CODAM (study, it has been found that the rs3134945 SNP of *RAGE *is associated with higher glucose levels in diabetics. As well, the G28S polymorphism of RAGE has shown to be associated with diabetic nephropathy (21). It was reported that *RAGE* promoter polymorphism and -374T/A variant have a protective effect against vascular complications (21).

***Protein kinase C activation pathway***

PKCs consist of at least 11 isoforms in mammalian tissues. Increased activation of several PKC isoforms is the third common pathway which mediates tissue injury through hyperglycemia-induced ROS. Increased ROS inhibits activity of the glycolytic enzyme glyceraldehyde 3-phosphate dehydrogenase (GAPDH), and increases the intracellular level of DAG precursor. Triose phosphate can enhance *de novo* synthesis of DAG from glucose. Interaction between AGEs and their cell-surface receptors an also increase the activity of PKC isoforms (Figure 2). It has been observed that the over-activity of PKC is implicated in decreased nitric oxide (NO) production in smooth muscle cells, and it has also shown to inhibit insulin-stimulated expression of endothelial NOS (eNOS) in cultured endothelial cells (9). In cultured mesangial cells of diabetic rats, expression of transforming growth factor (TGF)–β1 is induced and as a result, the microvascular matrix proteins are increased by abnormal activation of PKC β and δ isoforms. Besides, in cultured endothelial cells and vascular smooth muscle cells, hyperglycemia-induced activation of PKC can cause over-expression of plasminogen activator inhibitor-1* (PAI-1)* and the activation of NF-κB (18).This could result in vascular damage via inflammation; increases the permeability of basement membrane thickening, angiogenesis and thrombotic vascular occlusion.

***Hexosamine*** ***pathway***

Increasing the flux of fructose 6-phophate into the hexosamine pathway can also contribute to the pathogenesis of diabetic complications. Fructose 6-phosphate is diverted from glycolysis to provide glutamine fructose 6-phosphate amidotransferase (GFAT). After conversion of fructose 6-phosphate to glucosamine 6-phosphate by GFAT, it is converted into uridine diphosphate N-acetylglucosamine (UDP-N-acetylglucosamine). It has been shown that hyperglycemia causes a 4-fold increase in UDP-N-acetylglucosamine, which mediates hyperglycemia induced activation of the PAI-1 and TGF-β1. Normally, only a small percentage of glucose is metabolized through this pathway in which hyperglycemic condition is increased. It has shown that the increased flux of hexosamine pathway impairs insulin-signaling pathways (9). Other studies have shown that hyperglycemia mediated raising levels of both UDP-GlcNAc and O-GlcNAcylation lead to both oxidative and endoplasmic reticulum stress, which have caused chronic inflammation and insulin resistance (9, 13, 16) (Figure 2).

## Antioxidant genes and polymorphisms

***Glutathione peroxidase***

GPx is a seleno enzyme that exists within the cell and is involved in converting hydrogen peroxide (H2O2) and other organic peroxides into H2O and O2. Five isoforms of GPx; GPx 1-5 have been identified so far. The most studied isoform is *GPx1* and its Pro198Leu SNP is associated with various conditions such as cancer, diabetes, kidney and vascular disease. It has been revealed that T2DM patients with the Pro/Leu C/T polymorphism in *GPx-1* gene have shown increased macro-vascular measures such as carotid intima media thickness (CIMT), and also peripheral vascular disease compared to patients with Pro/Pro genotype. Nonetheless, no significant association was found between *GPx1* Pro198Leu SNP and myocardial infarction or stroke in T2DM patients. There was also no association between this SNP and diabetic neuropathy (DN) (2, 6).

***Catalase***

Catalase is a tetrameric hemoprotein which catalyzes the breakdown of H2O2 to H2O and O2. The -262C/T SNP is the most investigated SNP in relation to different types of diabetes including type 1, type 2, gestational diabetes, and diabetes’ complications such as retinopathy, nephropathy, ischemic heart disease, and cardiovascular disease (CVD) (2, 6). The CT+TT genotype of C111T variant of catalase has been shown to increase blood catalase activity in T2DM patients (22). Moreover, *CAT *-262C/T polymorphism was found to contribute to hypertriglyceridemia in Chinese T2DM or diabetic CVD patients (23). Although, it was shown in a study that there was no association between -262C/T polymorphism of *CAT* and diabetic retinopathy, nephropathy or ischemic heart disease in Caucasian T2DM patients (24).

In some studies no association was found between catalase -262C/T genotype and the risk of T1DM (6, 25). Conversely, in other studies it was shown that the catalase C allele is associated with increased risk of DN (6, 26, 27).

Investigating acatalasemia showed that in T2DM patients, the frequency of catalase gene mutations is increased. It has also been shown that catalase deficiency causes increased risk of T2DM. In addition hypocatalasemia, which results in low levels of SOD and GPx, can increase oxidative stress (3).

Catalase activity decreases under certain oxidative stress conditions as a result of conversion of cystein to cysteic acid and nitration of catalase. Heterozygote form of -21T/A polymorphism of catalase has also been found to increase the risk of T2DM in north Indians (2).

***Glutathione S transferase***

A decrease in GSH level in diabetic patients would make the cells more sensitive to oxidative stress because GSH plays a role against oxidative stress by scavenging free radicals and reactive oxygen intermediates (28).

GSTs are included in a family of metabolic enzymes which protect cells from oxidative damage by conjugating toxic and carcinogenic compounds to glutathione and thus catalyzing the detoxifying reaction. *GSTM1*, *GSTT1* and *GSTP1* are the most important genes of this family that are associated with an increased risk of T2DM (29-31).

Detoxification of genotoxins is catalyzed by GSTM1 class, while GSTT1 is involved in utilizing oxidized lipids and oxidized DNA. The products formed from DNA oxidation are catalyzed and detoxified by GSTP1 (28).

Several ethnic population-based studies have been designed to assess the association between the above four polymorphisms and T2DM. Although, controversial results have been found. Deletion of *GSTT1* was shown to be significantly associated with T2DM in Chinese and Egyptian populations. It was shown that null genotype of *GSTM1* and heterozygous genotype I/V of *GSTP1* were significantly associated with increased risk of T2DM in North Indian population. This significant association was also observed with the combination of the two high-risk genotypes including *GSTM1* null/*GSTT1* null, *GSTM1* null and *GSTP1* (I/I) (28). In South Iranian population, the null genotype of *GSTM1* was found to be associated with T2DM but neither *GSTT1* nor *GSTP1* were shown to be associated with the disease. yet, the combination of *GSTM1*-null and *GSTT1*-null genotype showed the increased risk of developing T2DM (29). In a case-control study in a Turkish population, it was shown that *GSTM1* null genotype and heterozygous genotype of *OGG1* Ser326Cys were significantly more frequent in T2DM patients. Additionally, the combination of null *GSTM1* and *OGG1*, null *GSTT1* and *OGG1* and *GSTM1*, *GSTT1* and *GSTP1* was revealed to be significantly associated with increased risk of diabetes in Turkish population (30). In another study, a significant association between *GSTM1* polymorphism, *GSTT1* polymorphism and T2DM was observed. Though, no association between *GSTP1* polymorphism and T2DM was found [31].

Three meta-analysis studies have been found which have assessed the association of *GSTM1* and *GSTT1* variants with T2DM and its complications (32-34) (Table 1). Yi et al. (32) and Zhang et al. (33) showed significant associations between individuals or combined effect of *GSTM1* or *GSTT1* null genotypes and diabetes risk. Orlewski et al. (34) found the influence of *GSTM1* null and combination of *GSTM1* null/*GSTT1* null genotypes in DN.

***NOS***

Nitric oxide via regulation of endothelial function and vascular tone inhibits aggregation of platelet, adhesion of leukocytes to vessels endothelium, and oxidation of low density lipoprotein cholesterol (LDL-C). Endothelial dysfunction secondary to NO production impairment results in insulin resistance, T2DM, chronic renal failure and cardiovascular complications manifesting hypertension and hypercholesterolemia (35).

NOS includes 3 isoforms; inducible nitric oxide synthase (NOSI/iNOS), neural NOS (NOSII/nNOS) and endothelial NOS (NOSIII/eNOS). Of the above isoforms, eNOS has been found to have a critical role in hypertension, DM, hypercholesterolemia and atherosclerosis (35). There is some evidence that several SNPs of eNOS have association with cardiovascular and kidney diseases which include T-786C, G894T (Glu298Asp) and 27bp-VNTR (variable number tandem repeat). In T2DM patients, *eNOS* Glu298Asp SNP can interact with different variations of other endogenous antioxidant enzymes. Since in T2DM patients, no association of *eNOS *Glu298Asp, *eNOS 4a/b* and *iNOS* Ser608Leu polymorphisms with DN was found, eNOS reduction might be considered as a biomarker of oxidative stress (2). There were several lines of evidence suggesting that G894T polymorphism of *eNOS* has no impact on the basal NO activity in renal circulation, while the T allele was associated with increased oxidative stress in the renal circulation in patients with diabetes (36).

In addition, *eNOS* haplotype has shown association with retinopathy in T1DM (37), but the association was controversial in T2DM (38, 39). Although ‘a’ allele of *eNOS* polymorphisms has shown a significant association with neuropathy in T2DM, but the association was more significant in diabetic patients who had no other complications (39).

The expression of *iNOS* in vascular smooth muscle cells, macrophages, and monocytes following exposure to inflammatory cytokines was found in atherosclerosis (2).

Since meta-analysis studies have been established as the highest rank in evidence-based medicine, we are reporting the results of meta-analysis studies performed in the association of *eNOS* gene variants and T2DM risk. We found one study for association with G894T, 4b/a, seven studies on T-786C variant and T2DM and four meta-analysis studies on this topic between these polymorphisms and DN and diabetic retinopathy (DR) (40-51) (Table 1). All eight meta-analysis studies that assessed the influence of *eNOS* gene variants on T2DM or DN risk revealed the positive association (40-47). Still, the results obtained from meta-analysis studies on DR risk (48-51) showed non-significant association between 4b/a polymorphism and DR.

According to the above-mentioned findings, biochemical compounds enhancing NO production from eNOS might be considered as novel therapeutic approaches to prevent or reverse vascular damages resulting from oxidative stress. Some of these compounds that have been identified in recent years include PKC inhibitor midostaurin, the eNOS enhancing compounds AVE9488 and AVE3085, and the polyphenolic phytoalexin trans-resveratrol (52).

***SOD***

SOD is responsible for converting superoxide (O^2-^) to H2O2. SOD includes 3 isoforms; SOD1 or CuZn-SOD (intracellular), SOD2 or MnSOD (mitochondrial), and SOD3 or EC-SOD (extracellular) enzymes (10).

SOD2 is a critical defender against mitochondrial superoxide radicals. The C47T (Val16Ala) polymorphism is the most important polymorphism in *SOD2* because this polymorphism can facilitate the transport of SOD2 into the mitochondrial matrix and increase the superoxide radicals neutralization (53).

Several studies have investigated the relationship between *SOD2* genotypes and diabetes’ complications. A study that investigated the association between SOD2 and kidney diseases has shown a positive association between *SOD2* Ala/Val and Val/Val genotypes and both CVD and diabetes (10).

Association between *SOD2* Ala allele and DR was shown in some studies (54, 55). But, in another study, Val/Val genotype was associated with DR controversially (56). In addition, in one study it was shown that the Val/Val genotype was associated with an increased risk of DN in both T1DM and T2DM (6).

Investigating *SOD2* gene A16V (C/T) polymorphism, it appeared that among T2DM patients, with and without macroangiopathy, DR in Chinese population, showed a significant difference in allele and genotype frequencies (2). Two meta-analyses showed that the C allele of C47T polymorphism (rs4880) of *SOD2* gene was significantly associated with reduced risk of DM; type 1 and T2DM, DN, diabetic neuropathy, and DR (53, 57) (Table 1).

**Table 1:** Association between antioxidant gene variants and T2DM or its complications in meta-analysis’ studies

**Gene**	**Variant(s)**	**Sample size (case/control)**	**Disease risk**	**P Value of association**	**Ref.**
**GST**	*GSTM1(* *rs4025935)* *GSTM1/GSTT1(rs4025935/* *rs71748309)* *GSTM1 (rs4025935)* *GSTM1-GSTT1 (rs4025935-rs71748309)* *GSTT1 (rs71748309)* *GSTP1*	2568/4486 2577/4572 874/ 966 874/ 966 874/ 966 874/ 966	DM DM DN DN DN DN	Sig. Sig. Sig. Sig. Non-Sig. Non-Sig.	[32] [33] [34] [34] [34] [34]
**eNOS**	*4b/a* *(rs3138808)*	4966/3043 2663/2232 2847/4268 6144/4900 2134/2348 3793/3161 1250/1368 3227/3437 3183/3410 3924/4187	T2DM DN DN DN DN DN DN DR DR DR	Sig. Sig. Sig. Sig. Sig. Sig. Non-Sig. Non-Sig. Non-Sig. Sig. Protective	[40] [41] [42] [43] [43] [45] [46] [48] [49] [50]
	*G894T* ***(*** *rs1799983)*	4795/3805 1942/1461 2654/1993 3924/4187 519/747	T2DM DN DN DR DR	Sig. Sig. Sig. Non-Sig. Non-Sig.	[40] [41] [45] [50] [51]
	*T-786C* *(rs2070744)*	875/845 2847/4268 1348/1175 273/450 1473/1572 3924/4187	DN DN DN DN DN DR	Sig. Sig. Sig. Non-Sig. Sig. Non-Sig.	[41] [42] [45] [46] [47] [50]
	*G986T*	2847/4268 850/1254	DN DN	Sig. Sig.	[42] [44]
	*rs41322052*	519/747 273/450 519/747	DR DN DR	Non-Sig. Non-Sig. Non-Sig.	[51] [46] [51]
**SOD**	*C47T* *(Val16Ala)* *(rs4880)*	2454/1901 2454/1901 2454/1901 2454/1901 119/875	DM DN DR DMI DN	Non-Sig. Protective Sig. Protective Sig. Protective Sig. Protective Sig. Protective	[53] [53] [53] [53] [57]
**NOX**	*C242T* *(rs4673)*	1661/1265 1068/1026 486/273	T2DM DN CA	Sig. Sig. Non-Sig.	[65] [66] [66]

**Legends:** **GST**, glutathion-S-transferase;** DM, **diabetes mellitus;** Sig, **significant; **Non-Sig**., non-significant; ** DN**, diabetic nephropathy;** eNOS**, endothelial nitric oxide synthse; **T2DM**, type 2 diabetic mellitus;** DR**, diabetic retinopathy, **SOD**, superoxide dismutase;** DMI**, diabetes microvascular, **NOX**, nicotinamide adenine dinucleotide phosphate (NADPH) oxidase; **CA**, carotid atherosclerosis.

***NADPH oxidase (NOX)***

NOX consists of seven isoforms; NOX1-5, DUOX (dual oxidase) 1 and DUOX2 (58, 59). The main function of NOX is to produce ROS. NOX-derived ROS appears to modulate redox-sensitive mitogen-activated protein (MAP) kinases (extracellular signal-regulated kinases (ERK1/2) , p38MAP kinase, c-Jun N-terminal kinases (JNK)), pro-inflammatory kinases (ERK5), involved in protein synthesis, cell cycle progression and cell proliferation, tyrosine kinases (c-SRC, epidermal growth factor receptor (EGFR), phosphoinositide 3-kinase (PI3K) ) and transcription factors including those that have been extensively linked to inflammation (NF-κB, AP-1 and hypoxia-inducible factors (HIF-1)) (1). Thus, part of NOX-derived ROS roles could be in gene expression, apoptosis, cell growth, and oxygen sensing (60).

It is demonstrated that NOX has a key role in the regulation of normal renal function via regulation of its blood flow, alteration of cell fate, and regulation of gene expression. Under pathological conditions such as oxidative stress, NOX-derived ROS can result in vasoconstriction, tissue fibrosis, inflammation, and impaired vascular and renal function (60). Main stimulators of the activation of NOX enzymes are angiotensin II and aldosterone. Within NOX isoforms, mostly NOX-4 and partially NOX2 have some key roles in pathology of kidney diseases. Increased formation of ROS has been demonstrated in other endothelial cells such as cardiomyocytes (60).

In Nox1-/y mice, a predominant role for Nox1-derived ROS was demonstrated in relation to diabetes-associated atherosclerosis (1).

In T2DM mice, the enhancement of *Nox2* expression and decreased ROS production by AGE blockers were shown. Thus, it seems that Nox oxides have a critical role in mediating the effects of AGEs (59).

In cultured Bovine retinal pericytes, the increased expression of *Nox2* was observed which caused on increase in apoptosis of these cells suggesting the pathological role of Nox2 in DR (61). likewise, in diabetic mice treated with streptozotocin, upregulation of *Nox2* gene in the kidneys was observed indicating the role of Nox2 in the development of renal oxidative stress and eventually DN (58).

There is no report with regard to the association between Nox3 or Duox1/Duox2 and diabetic complications (59).

There is evidence of association between Nox4 and DN (59). In addition, increased expression of *Nox4* is found in myocardial and aorta of T1DM and T2DM mice (59). Hence, Nox4 siRNA or Nox1/4 inhibitor might be able to block the effect of hyperglycemia on deposition of fibronectin in kidney. Improvement of renal function by Nox1/4 inhibitor was illustrated in an animal-model of diabetes that was independent of glucose control (58). In mouse models, in the proximal and distal tubules, it appeared that *Nox4 *expression and eventually ROS generation increased. Some studies showed that *Nox4 *knockout mouse models are less prone to develop DN in contrast to others (1, 62, 63). Increased expression of *Nox4* and vascular endothelial growth factor (*Vegf*) was observed in animal model of T2DM in the retina. Besides, exposure of bovine retinal endothelial cells to high glucose revealed the same results suggesting critical role of Nox4 in the vascular dysfunction of DR (61). Similar to AGE pathway, PKC and Rho/Rho kinase can be considered to control pathways of Nox4 (59).

Studies have exhibited the existence of AP-1, NF-κB and (signal transducer and activator of transcription 1/3 (STAT1/ STAT3) regulatory sites in the promoter region of *Nox5*, and demonstrated that all of these sites are involved in the development of vascular complication in diabetes. In a transgenic mouse model which expressed *Nox5* in podocytes, it has been discovered that Nox5 is involved in DN (1).

Along with this, over - activation of NADPH enzymes can induce insulin resistance that is confirmed by a study in cultured L6 myotubes (64).

Two meta-analysis studies have assessed the association of C242T **(**rs4673) variant of NADPH oxidase *P22-*P*HOX *gene with T2DM, DN and carotid atherosclerosis (CA) risk (65-66). In these studies, a significant association between C242T polymorphism and T2DM or DN was demons trated but the association between this variant and CA was non-significant (Table 1).

Considering the role of NOX in ROS generation and diabetic complications, NOX inhibitors such as apocynin could be used as therapeutic compounds for treatment of various diabetic complications, such as nephropathy, retinopathy and cardiovascular diseases (1, 58, 59).

**Conclusions**

Altogether, it has been shown that genetic markers can be used in the prediction and diagnosis of disease-related factors and are helpful in unraveling the pathophysiology of various conditions including T2DM. There is a great consensus that several other alterations including epigenetic variations might also be involved in the development of disorders besides genetic variations, which need to be taken into account while interpreting the genetic association data with controversial results. As a result, future studies should be directed such that both genetic and epigenetic variations as a network of various outcomes in the disease progression, be elucidated. In this vein, there is a great requirement for designing further studies to examine the involvement of other factors including epigenetic markers in T2DM.
